# Using Electronic Health Records for the Learning Health System: Creation of a Diabetes Research Registry

**DOI:** 10.2196/39746

**Published:** 2022-09-23

**Authors:** Brian J Wells, Stephen M Downs, Brian Ostasiewski

**Affiliations:** 1 Department of Biostatistics and Data Science Wake Forest University School of Medicine Winston Salem, NC United States; 2 Department of Pediatrics Wake Forest University School of Medicine Winston Salem, NC United States; 3 Center for Biomedical Informatics Wake Forest University School of Medicine Winston Salem, NC United States

**Keywords:** electronic health record, EHR, Learning Health System, registry, diabetes

## Abstract

Electronic health records (EHRs) were originally developed for clinical care and billing. As such, the data are not collected, organized, and curated in a fashion that is optimized for secondary use to support the Learning Health System. Population health registries provide tools to support quality improvement. These tools are generally integrated with the live EHR, are intended to use a minimum of computing resources, and may not be appropriate for some research projects. Researchers may require different electronic phenotypes and variable definitions from those typically used for population health, and these definitions may vary from study to study. Establishing a formal registry that is mapped to the Observation Medical Outcomes Partnership common data model provides an opportunity to add custom mappings and more easily share these with other institutions. Performing preprocessing tasks such as data cleaning, calculation of risk scores, time-to-event analysis, imputation, and transforming data into a format for statistical analyses will improve efficiency and make the data easier to use for investigators. Research registries that are maintained outside the EHR also have the luxury of using significant computational resources without jeopardizing clinical care data. This paper describes a virtual Diabetes Registry at Atrium Health Wake Forest Baptist and the plan for its continued development.

## Background

The first electronic health records (EHRs) were developed to support clinical care, but later became primarily focused on billing after the creation of diagnosis-related group (DRG) codes [1]. DRGs are intended to provide precise estimates of resource use across different hospitals. Unfortunately, the documentation necessary to support billing frequently does not result in a data content and structure ideal for the secondary use of these data for research. Safran et al [2] outlined a framework for using EHR data for secondary purposes. The use of EHR data for research purposes has increased significantly at Wake Forest and elsewhere over the past several years. However, complex outcome studies that use data at different time points are still rare. Research investigators struggle with the processing and statistical analyses of EHR-derived data due to the time-varying nature, inconsistency, inaccuracy, lack of documentation, and incompleteness of clinical data. Investigators report that the amount of time spent deciphering and *cleaning* these data make many research projects impractical. A systematic review of the use of EHR data for population health identified several common barriers for the use of these data for population health, of which missing data were most cited [3]. Handling of missing data requires an understanding of the reasons for missing data, some of which can be project-specific reasons and related to decisions about how to handle them. Simply excluding patients with missing data may reduce sample size and can lead to biased results. One common method for handling missing data in EHR projects is multiple imputation, where statistical models are used to estimate values for missing data elements [4]. Investigators may be unfamiliar with these techniques or may lack the knowledge and skills to perform the task in a robust fashion. Imputation will be one of the services provided to investigators. Prior to imputation, it is necessary to explore data to identify implausible values that may arise due to inaccurate measurements or data entry errors. Textbox 1 highlights some of the data-processing steps that may be required prior to using clinical data for statistical analyses.

**Common data-processing steps required to analyze clinical data.**

**Common data-processing steps**

- Removal of extreme values
- Correction of erroneous entries
- Imputation of missing values
- Calculation of predefined variables
- Determination of active medication classes on a given date
- Calculation of dates and time to events
- Creation of a single analytic data set with a single row per patient from normalized tables

### Research Registries

Research registries derived from the EHR can provide a foundation that improves the efficiency for research projects in a specific disease area. Registries can provide formal documentation of the institutional knowledge gained over time from previous investigations and input from the research community. The sharing of experiences provides an opportunity for critical evaluation of the data from investigators with different areas of expertise, leading to improved data quality and knowledge of the data necessary for interinstitutional projects. Preprocessed data, predefined variables, linkage with other institutional databases (eg, echocardiogram and pulmonary function tests), linkage with external data (eg, American Community Survey and North Carolina Death Registry), and creation of statistical functions can greatly reduce the time and cost of secondary data analyses. Data preprocessing can include data cleaning (eg, removal of extreme values and imputation of missing data), which can reduce the risk of biased results but would be inappropriate for clinical data. Prescription medications provide another opportunity for data preprocessing. For example, calculation of dosages and quantity of medications can be determined by applying regular expressions to free text prescription instructions. Research registries also provide a mechanism for pooling knowledge and resources from disparate research areas. For example, chart reviews conducted for one specific research study could provide important knowledge that benefits all users of the registry. Similarly, researchers could pool resources to purchase external data (eg, National Death Index or Centers for Medicare & Medicaid Services [CMS] data) that will benefit all. Research registries provide a repository for collecting research items not intended for the legal medical record to support activities such as creating risk prediction models and conducting epidemiologic studies. Furthermore, the research registries also provide potential populations of patients for research studies (clinical trials, pragmatic trials, implementation science, population health, and medical informatics). The increased recognition and credibility of an institution’s clinical data for research that comes with a successful registry can improve the chances for research funding.

## Population Health Registries

There has been a proliferation of population health registries in EHR systems. These real-time data are necessary for clinical care, and these registries are designed to put minimal burden on the EHR system, especially given that they are using the live EHR system, which is critical for clinical care. These types of EHR-based population health registry tools (eg, Healthy Planet, Epic Systems) provide current snapshots of patients and are helpful for population health management. These operational reporting tools are fast, provide real-time data, and are incorporated into the clinical workflow. These minute-by-minute updates of clinical data are unnecessary for many types of secondary data analyses. Population health registries have motivations that may differ from research investigations. For example, population health registries support quality-based metrics such as indicators maintained by the National Quality Foundation, which may be publicly reported and are used to guide reimbursement incentives for programs such as the Medicare Shared Savings Plan. In these instances, disease phenotypes and variable definitions are pre-defined by the interested parties. In this scenario, there may be a single criterion used to define the population and associated metrics. Creating additional criteria would be counterproductive. By contrast, a research registry should provide comprehensive data on members collected over time, requires statistical analyses, and may contain multiple definitions for the same variable. These data allow evaluations at user-defined time points or time-varying analyses. Because the tool is not integrated into clinical workflows, there is an opportunity to incorporate large quantities of data into computationally intensive analyses that would otherwise be a drain on clinical systems.

Population health registries are ideally suited for clinical care and quality improvement in that they are available instantaneously on the live EHR, have standardized definitions, and use limited computing resources. By contrast, the type of research registry that we have created enables the creation of different cohorts for the same disease entities, makes use of additional computation resources that would be inappropriate for the clinical EHR and allows different variable definitions depending on the specific study. Table 1 lists additional differences between our research registry and population health registries.

Registries created from EHR data may have different goals and requirements. The table compares features of research and population health registries.

It should also be noted that EHR vendors each use their own proprietary *technical* data models that will map to ontologies such as International Classification of Diseases codes. The precise mappings are not made publicly available, which makes multicenter studies involving different EHR systems more difficult. The registry we have built is mapped to the Observational Medical Outcomes Partnership (OMOP) common data model (CDM). CDMs such as OMOP have been instrumental in creating interoperability standards in support of clinical research networks that span multiple institutions. This registry will take advantage of the data mappings available in OMOP and benefit from the automated tools developed for OMOP for identifying potential data issues. The Phenotype Knowledge Base contains a repository of electronic phenotypes to support registry construction and variable definitions [5]. These phenotypes have been successfully integrated into the OMOP data model to facilitate implementation at different research institutions [6]. We will also have the opportunity to create additional custom mappings to our OMOP instance, which can be leveraged by local researchers.

**Table 1:** Characteristics of research registries vs population health registries.

Research registry	Population health registry
Intermittent updates	Real-time updates
Higher computational resources	Low resource use
Complex definitions from a variety of sources and multiple definitions for similar concepts	Simple definitions defined by QI-based^a^ reimbursement
Variety of external data sources	Data limited to EHR^b^
Extensive data processing	Limited data processing
Complex temporal relationships	Single point in time
Easily accessible and detailed documentation	Documentation or coding sometimes lacking or not easily accessible
Does not need to be integrated into workflow	Integration in clinic workflow is crucial
Does not require front-end EHR access.	Requires front-end EHR access with PHI^c^
Mapped to open-source common data models	Mapped to vendor-based *technical* data models

^a^QI: quality improvement.

^b^EHR: electronic health record.

^c^PHI: protected health information.

## Custom Phenotypes

**Figure 1:**
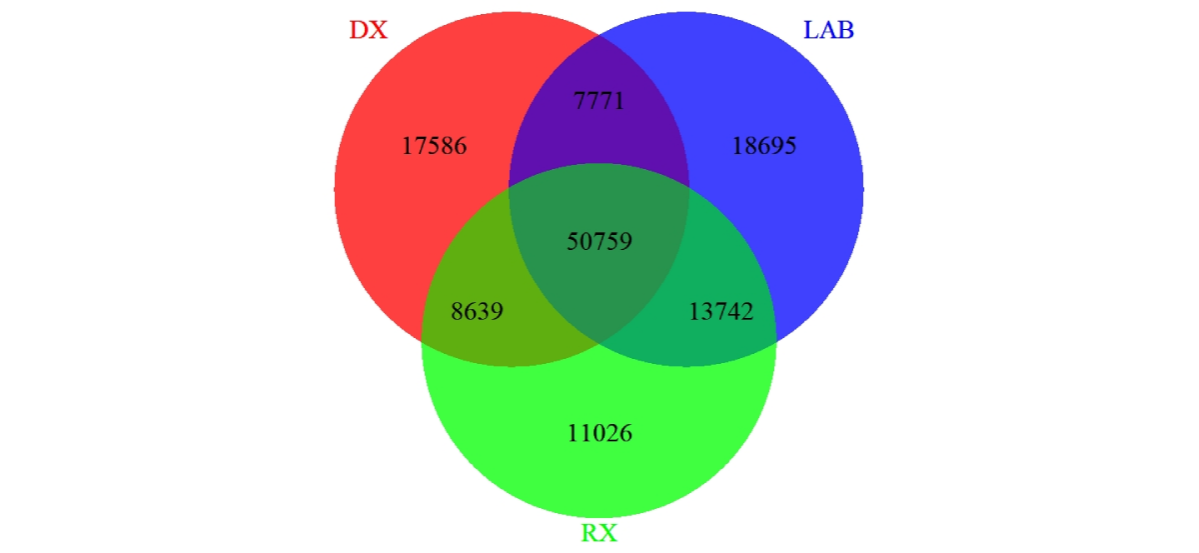
Sets of patients with possible diabetes according to definitions based on diagnoses codes (DX), laboratory values (LAB), or prescriptions (RX).

As mentioned previously, research projects may require variable definitions that are different from quality-based metrics, and variable definitions may vary from one project to the next. Varying variable definitions are also necessary for cohort discovery. The definition of diabetes may differ between projects. For example, a case control study needing a limited number of cases may want to have a highly specific definition for type 2 diabetes such as the one created by Kho [7]. By contrast, a study evaluating the accuracy of different electronic phenotypes may require a highly sensitive definition to capture all possible diabetes cases for manual chart review [8]. Figure 1 shows a Venn diagram illustrating the different patient populations that would be captured from our data warehouse depending on whether one uses diagnosis codes, hemoglobin A_1c_ laboratory values, or prescriptions for hypoglycemic medications.

In other instances, existing definitions may be available from agencies such as Agency for Healthcare Research and Quality or the CMS. For example, we used the CMS definition for an acute exacerbation of chronic obstructive pulmonary disease for a study looking at the impact of a chronic obstructive pulmonary disease care pathway on reducing readmissions [9].

In addition to phenotypes used for cohort discovery, research projects require definitions for covariates included in the statistical analyses. Depending on the situation, investigators may desire different definitions for comorbidities such as hypertension. Textbox 2 shows the contrast between an example of a simple definition for hypertension based on diagnoses codes vs a complex definition that might be used for a study, where maximizing the sensitivity for identifying hypertension is key.

**Example definitions of hypertension.**

**Research registry**

- International Classification of Diseases (ICD) code for hypertension (HTN) in encounter diagnoses, past medical history, or problem list *OR*
- Minimum of 3 blood pressure (BP) readings >140/90 over 3 months in the electronic health records
  - Outpatient BP excluding urgent care clinics, emergency department, or observation visits
  - Based on last BP of encounter
  - Exclude BPs when associated temperature≥38 °C *OR*
- Active prescription for an antihypertensive agent

**Population health**

- ICD code for HTN in encounter diagnoses

## OMOP Limitations

While the use of OMOP has many advantages in terms of standardization, there are still significant areas of limitations. Medications are one area where common data models are still lacking. For example, OMOP contains a single drug exposure table for prescriptions, drug administration, dispensing information, and patient-reported information. Unfortunately, dispensing information, patient-reported information, and compliance are rarely captured in structured EHR data. In addition, there are no explicitly linked medical reasons for the exposures in OMOP, and the RxNorm categorizations may not be appropriate for a specific research study. A registry cannot resolve all these issues, but the structure provides the flexibility to create and validate new phenotypes. For example, researchers can create and share relevant medication groupings, and algorithms based on specific prescription information (eg, dates of prescriptions, stop dates, number of pills, and number of refills) can be created as proxies for active medications and compliance. Similarly, associated information (eg, presence or absence of different diagnoses codes and laboratory values) can define reason for medication. These new phenotypes can be used locally and shared with the OMOP community without being formally integrated into the OMOP model.

OMOP will not be able to represent all the new phenotypes that the registry will require, making it necessary to characterize our own concepts. Some of these concepts may be derived entirely from existing OMOP concepts, but many will require the creation of our own. Like all CDMs, OMOP has limitations in its capacity to represent information inherent to the transformation from one data model (eg, EHR) to another. In addition, it will be crucial to have a formal data quality structure in place to ensure mappings are correct and routinely updated as data change. We have established a phenotype working group that includes the authors as well as additional faculty members in the Center for Biomedical Informatics.

## Data Structure

**Figure 2:**
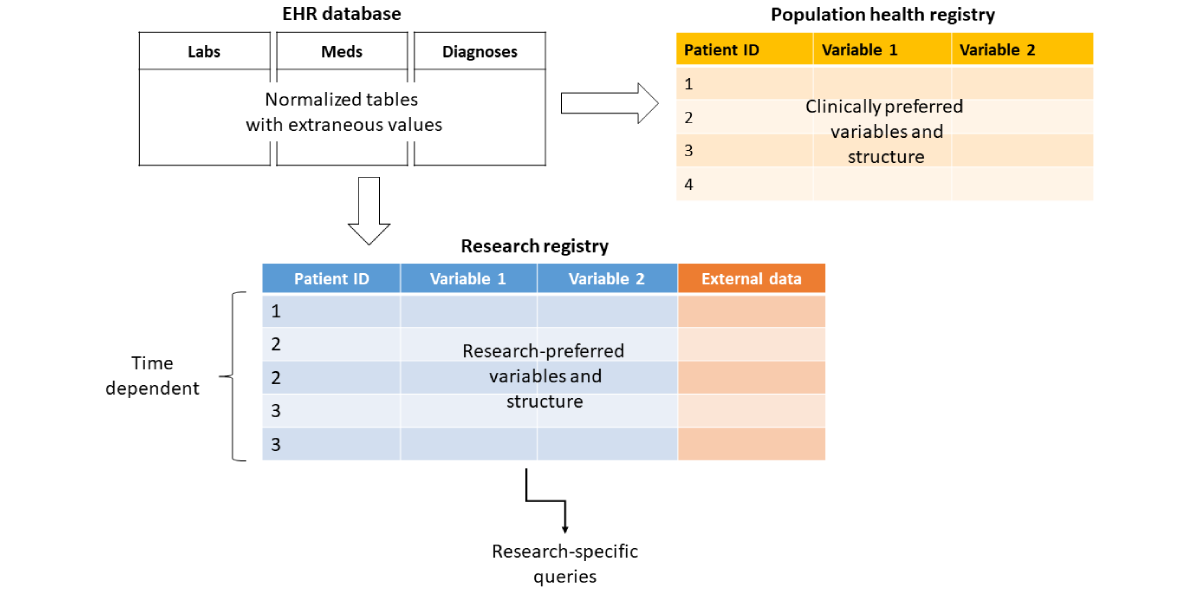
Comparing data structures of electronic health records (EHR), population health registries, and research registries.

The data structure of the EHR database, a typical population health registry, and a research registry can vary significantly. EHR databases are stored in database management systems using individual, partially normalized tables for each specific data domain. This structure reduces storage space and speeds data extractions. By contrast, most statistical analyses require an individual flat data table (also known as *pivot table*), where the unit of analyses is the individual rows of patients. The data sets need to include columns for both independent and dependent variables and may require calculations of follow-up time between baseline variables and the outcomes of interest. Some external variables that do not exist in the EHR may be linked with the data set. For example, we link our registry with the North Carolina state death index, allowing better ascertainment of mortality outcomes and censoring of follow-up time. Variables may also be derived from the source data (eg, highest blood pressure in the past 24 hours) and time dependent analyses necessitate multiple rows for each patient that reflect the patient’s current state at a given point in time. Figure 2 provides a graphical representation of the different data structures between the EHR database, an EHR-based population health registry, and a research registry.

## Diabetes-Specific Registry

We chose diabetes as one of the first registries to make available in our Clinical and Translational Science Award Program given that it represents a focus area of our research enterprise. In addition, diabetes is a natural choice for a research registry given the rising incidence, chronic nature, established quality metrics, comorbidities, availability of treatments, and research funding. Research also indicates that blood sugar and associated risk factors are poorly controlled in patients with diabetes. In addition to a desire for improving the health of their patients, health care institutions have direct financial incentives for adequately treating patients with diabetes. Quality indicators approved by a successful diabetes research registry would provide an opportunity for the creation of risk prediction models that could be used to target patients at high risk as well as those who are most likely to benefit from a specific intervention. Thorough statistical evaluations of quality improvement projects and population health interventions would provide crucial feedback on the potential net benefits of these programs.

**Figure 3:**
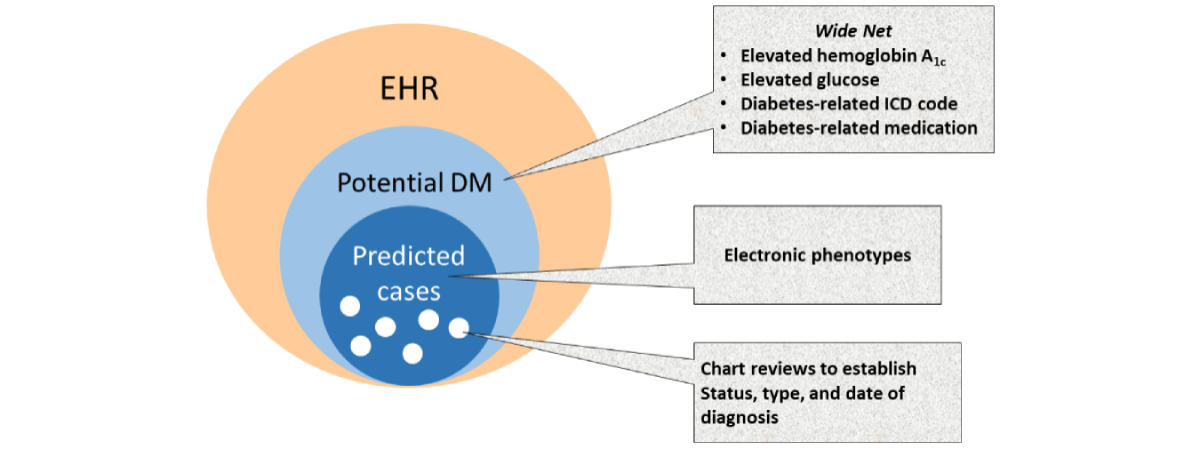
Venn diagram showing the use of electronic algorithms combined with chart reviews to identify patients with diabetes. DM: Diabetes Mellitus; EHR: electronic health record; HbA_1c_: hemoglobin A_1c_; ICD: International Classification of Diseases.

The identification of diabetes in EHR is surprisingly complex. Common methods for identifying potential cases include searches for medications, laboratory values, and diagnosis codes. Each of these approaches has its own limitations. Medications used for diabetes may also be used to treat other conditions. For example, metformin is commonly prescribed for polycystic ovarian syndrome in women. Blood glucose values may be abnormally elevated due to inadequate fasting times, which are generally not easily determined in the EHR. Diagnosis codes may be incorrectly used before patients meet formal criteria for diabetes or may be associated with the incorrect diabetes type. The issues in correctly identifying patients with diabetes highlight the importance of flexible research registries. Recognizing the potential need for different diabetes definitions, we chose to create our registry based on the concept of a highly sensitive *Wide Net* with the goal of capturing any evidence of possible diabetes in the EHR. Figure 3 provides a graphical display of this concept.

This approach mirrors the one used by the SEARCH for Diabetes in Youth evaluation of using EHRs for diabetes surveillance [8]. Approaches such as these are necessary given the infeasibility of manually reviewing all patient charts. The SEARCH work found that the simple use of diabetes codes could accurately determine EHR evidence of diabetes, and the ratio of type 1 to type 2 codes had a high sensitivity and specificity for identifying youth with type 1 diabetes. Additional work is needed to determine the accuracy of this approach in adults, and further algorithms are needed for identifying children with type 2 diabetes or other diabetes types. This registry provides a great source of data for future electronic phenotypic development and validation.

Our registry contains 128,218 patients with possible diabetes according to one or more of these 3 domains, while only 50,759 patients have evidence of possible diabetes based on all 3 variables simultaneously (Table 2). Identifying random subsets of patients who meet different combinations of these criteria provides an opportunity to glean valuable information from manual chart reviews of these patients. Annotated data sets allow for evaluation of existing and creation of new electronic phenotypes for diabetes status, type, and date of diagnoses.

**Table 2:** Characteristics of patients^a^ who showed evidence of possible diabetes based on diagnoses codes, laboratory values, or medications.

Characteristics			Cohort 1: diagnosis		Cohort 2: labs^b^		Cohort 3: medications
Total unique patients, n (%)			84,755 (66)		90,967 (71)		84,165 (66)
Age (years), median (IQR)			66.02 (19.43)		65.46 (20.20)		64.62 (20.98)
**Sex, n (%)**							
	Female	43,510 (51.34)		44,008 (48.38)		43,374(51.53)	
	Male	41,239 (48.66)		46,950 (51.61)		40,783 (48.46)	
**Race, n (%)**							
	White	59,547 (70.26)		65,693 (72.22)		60,014 (71.30)	
	Black	19,120 (22.56)		19,042 (20.93)		17,905 (21.27)	
	Other	5794 (6.84)		5938 (6.53)		6004 (7.13)	
	Missing	286 (0.34)		267 (0.29)		223 (0.26)	
Ever smoker, n (%)			43,414 (51.22)		48,842 (53.69)		44,133 (52.44)
Insulin (1 or more prescriptions in the past year), n (%)			25,663 (30.28)		25,943 (28.52)		26,685 (31.70)
Charlson comorbidity index, n (median)			83,699 (2)		89,692 (2)		83,094 (2)
Median household income, n (median)			66,034 (46,283)		69,253 (45,688)		64,839 (45,927)
Most recent hemoglobin A_1c_, n (median)			64,959 (6.9)		72,833 (7.1)		69,933 (7.0)
Most recent eGFR^c^, n (median)			73,037 (70)		88,633 (66)		80,424 (70)
Most recent LDL^d^, n (median)			58,463 (88)		60,398 (88)		59,864 (89)

^a^Patients may exist in 1, 2, or all 3 of the cohorts.

^b^Random blood sugar ≥200 mg/dL or hemoglobin A_1c_≥6.5%.

^c^eGFR: estimated glomerular filtration rate calculated using the Chronic Kidney Disease Epidemiology Collaboration (CKD-Epi) equation.

^d^LDL: low-density lipoprotein.

## Jupyter Notebooks

Much like the interinstitutional heuristic and algorithm sharing enabled by sites supporting an OMOP CDM, there is potential for intrainstitutional collaboration and technique leveraging. Views in OMOP can be created by the honest brokers to provision only the cohort and relevant data permitted by an institutional review board application to specific authorized study personnel.

Jupyter is a free, open-source, interactive web-based computational notebook widely adopted by data scientists across thousands of enterprises, including Fortune 500 companies, international research facilities, universities, and start-ups. A Jupyter hub server allows users to centrally create and share codes, equations, visualizations, as well as text and results. It will also allow researchers to interact directly with their data views in OMOP via a programmatic language of their choice, whether it be Python (Python Software Foundation), R (The R Foundation), or even direct SQL. A library of Jupyter Notebooks with example code and outputs provided by data analysts can give researchers a rich starting base of programmatic techniques that they can modify, improve, and share back for other researchers to use in their own Jupyter Notebook analyses, greatly reducing the learning curve and lessening code redundancy and reimplementation.

## Schematic

**Figure 4:**
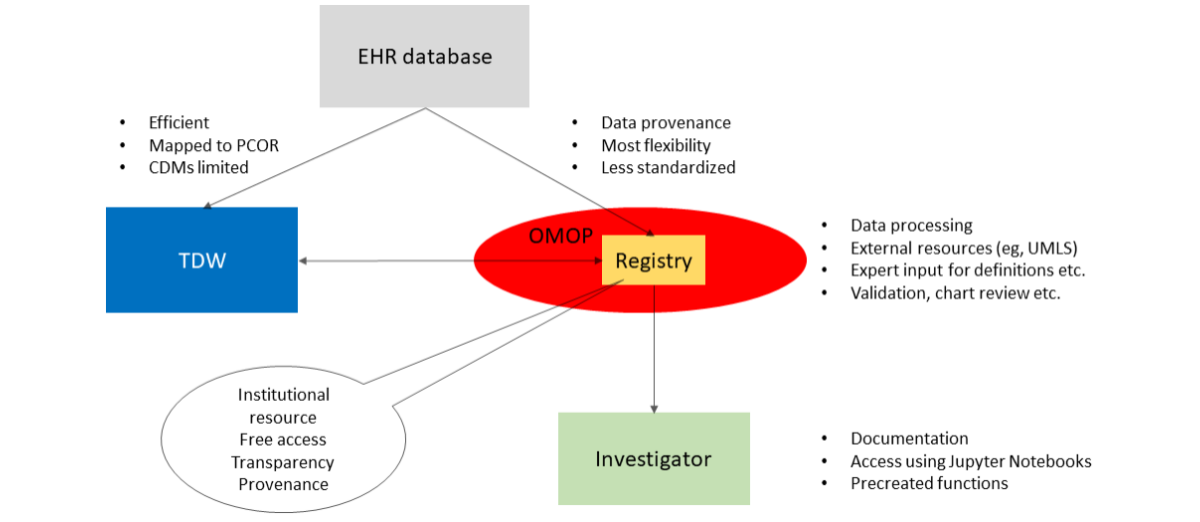
Schematic of the overall architecture of the registry, highlighting some of the guiding principles governing the registry creation. CDM: common data model; EHR: electronic health records; OMOP: Observational Medical Outcomes Partnership Common Data Model; PCOR: patient-centered outcomes research common data model; TDW: Translational Data Warehouse in the Wake Forest Clinical and Translational Science Institute; UMLS: Unified Medical Language System.

Figure 4 shows a schematic of the overall architecture of the registry and highlights some of the guiding principles governing the registry creation.

Data processing will undoubtedly uncover errors in the clinical data (eg, implausible values), which will be cleaned for data analyses. Data cleaning will be performed at the registry or post–data extract level. We are not attempting (at least at this point) to try and change values in the source clinical data, which is a difficult process and could have clinical implications. It is our hope that the registry could be used for data quality projects that might recognize a way to improve data collection or documentation.

As mentioned previously, the registry is mapped to the OMOP CDM and linked with our existing translational data warehouse. This ensures the standardization of data within the registry while exploiting our established infrastructure. Infusion of additional data from the vendor EHR database as well as data external to our Clinical Information Systems and our institution provides flexibility and continued creation of additional phenotypes. We have created a digital phenotype working group that will prioritize electronic phenotype creation and ensure appropriate documentation. Access to the registry through Jupyter Notebooks increases transparency and simplifies the sharing of code between investigators.

## Data Extracts

**Figure 5:**
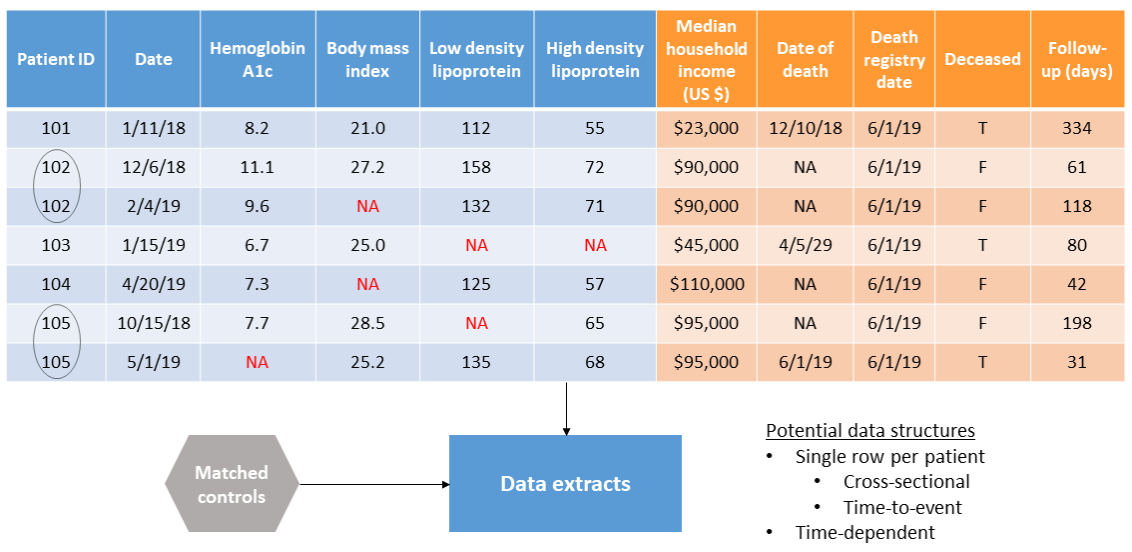
Example analytic data set extracted from the registry in a pivot table format. F: false; NA: not applicable; T: true.

Using existing R code created at Wake Forest will allow investigators to extract individual analytic tables that define patient characteristics at each given point in time per the specific study design. Figure 5 highlights how this table would appear.

Additionally, a Wake Forest Center for Biomedical Informatics–sponsored pilot grant is establishing a tool for creating randomly selected control patients to simplify the conduct of case control studies. We also have existing R code for the imputation of missing values using multiple imputation with chained equations that can be applied after the analytic data set has been created. Creation of multiply imputed data sets allows an estimation of the amount of missing information and stability of coefficient estimates [4].

## Future

Although the registry will be based on coded information, we recognize the growth in the data science community of graph representation of data. The ability to use Jupyter Notebooks to access data and to create and share code will allow investigators to integrate new methods such as graph theory for statistical analyses and to create data visualizations to share. We are particularly interested in examining diabetes-related treatment pathways and intend to use the concept relationship table in OMOP to define treatment pathways commonly used as well as pathways based on guidelines. The characterization of treatment pathways is ripe for graph representation.

We recognize that the data, informatics tools, and analytic techniques available for EHR-based analyses are rapidly changing. We have identified a group of clinical, informatics, and statistical professionals who can serve as registry stakeholders. Periodic meetings will allow for continuous feedback that will guide decisions on registry directions and priorities. The Wake Forest Clinical and Translational Science Institute has an established mechanism for continuous evaluation of the informatics program, of which this registry will be a part. Evaluations will include metrics on registry use, publications and grants using the registry, as well as formal (eg, surveys) and informal feedback.

## Summary

Secondary use of EHR data for research is still in its infancy, and tools to aid investigators in complex epidemiological-type studies needed for the Learning Health System are lacking. Typical population health registries do not provide the flexibility, computational resources, and data complexity necessary for many research endeavors. The virtual diabetes registry described in this paper is providing our researchers with tools that we hope will enable them to conduct sophisticated statistical analyses in the most transparent and efficient way possible. The registry is being built in a way that will allow for its continuous refinement based on user experience and in a format that will enable interinstitutional collaboration.

## Abbreviations

CDM: common data model
DRG: diagnosis-related group
EHR: electronic health record
OMOP: Observational Medical Outcomes Partnership
